# Multiple arboviral infections during a DENV-2 outbreak in Solomon Islands

**DOI:** 10.1186/s41182-020-00217-8

**Published:** 2020-05-15

**Authors:** Andrew Waleluma Darcy, Seiji Kanda, Tenneth Dalipanda, Cynthia Joshua, Takaki Shimono, Pheophet Lamaningao, Nobuyuki Mishima, Toshimasa Nishiyama

**Affiliations:** 1 Department of Hygiene and Public Health, Kansai Medical University, Hirakata, Japan; 2 Ministry of Health & Medical Services, Honiara, Solomon Islands

**Keywords:** Dengue fever, Arbovirus, Seroprevalence, Molecular, Immunity

## Abstract

**Background:**

Solomon Islands, a country made up of tropical islands, has suffered cyclic dengue fever (DF) outbreaks in the past three decades. An outbreak of dengue-like illness (DLI) that occurred in April 2016 prompted this study, which aimed to determine the population’s immunity status and identify the arboviruses circulating in the country.

**Methods:**

A household survey, involving 188 participants in two urban areas (Honiara and Gizo), and a parallel hospital-based clinical survey were conducted in April 2016. The latter was repeated in December after a surge in DLI cases. Arbovirus IgG ELISA were performed on the household blood samples to determine the prevalence of arboviruses in the community, while qPCR testing of the clinical samples was used to identify the circulating arboviruses. Dengue virus (DENV)-positive samples were further characterized by amplifying and sequencing the envelope gene.

**Results:**

The overall prevalence rates of DENV, Zika virus, and chikungunya virus were 83.4%, 7.6%, and 0.9%, respectively. The qPCR positivity rates of the clinical samples collected in April 2016 were as follows: DENV 39.6%, Zika virus 16.7%, and chikungunya virus 6.3%, which increased to 74%, 48%, and 20% respectively in December 2016. The displacement of the circulating serotype-3, genotype-1, with DENV serotype 2, genotype cosmopolitan was responsible for the outbreak in 2016.

**Conclusions:**

A DENV outbreak in Solomon Islands was caused by the introduction of a single serotype. The high prevalence of DENV provided transient cross-protection, which prevented the introduction of a new serotype from the hyperendemic region for at least 3 years. The severe outcomes seen in the recent outbreak probably resulted from changes in the causative viruses and the effects of population immunity and changes in the outbreak pattern. Solomon Islands needs to step up surveillance to include molecular tools, increase regional communication, and perform timely interventions.

## Introduction

Dengue fever (DF) is the most prevalent mosquito-borne viral infection in tropical and subtropical regions. It is estimated to cause 50–100 million symptomatic infections annually [1]. The complex interactions between the virus and the human population have resulted in an unpredictable disease evolution and variable clinical outcomes associated with the infection. The majority of infections are mild and self-limiting, but a small number progress to severe clinical manifestations, which can lead to severe organ complications and even death [2–4]. Dengue virus (DENV) is a *Flavivirus* from the *Flaviviridae* family. Its RNA genome is approximately 11-kb long and contains an open reading frame, which codes for three structural and five non-structural proteins. The virus has four distinct antigenic serotypes and several genotypes, which produce different outcomes during outbreaks [5–7].

Most global DF cases occur in the Asia-Pacific region [1]. Countries in Southeast Asia (SE Asia) are generally more urban and have high population densities, while the Pacific Island countries (PICTs) are rural, and their populations are scattered across many islands. Previous DF outbreaks in the PICTs were caused by a single serotype, which occurred every 5 to 10 years [8]. On the other hand, in SE Asia all four DENV serotypes are circulating, and a 3-year wave-like outbreak pattern, in which the disease emanates from a central metropolitan city, is seen. There have been very few quality descriptive studies of DF conducted in the PICTs, resulting in a paucity of information about the magnitudes and drivers of DF epidemics in this region. The available studies were mostly based in a clinic setting or involved specific groups like a blood donor population [9, 10]. The principal vectors of DF, *Aedes aegypti* (Linnaeus, 1762) and *Aedes albopictus* (Skuse, 1894), are present in both SE Asia and the PICTs.

**Fig. 1:**
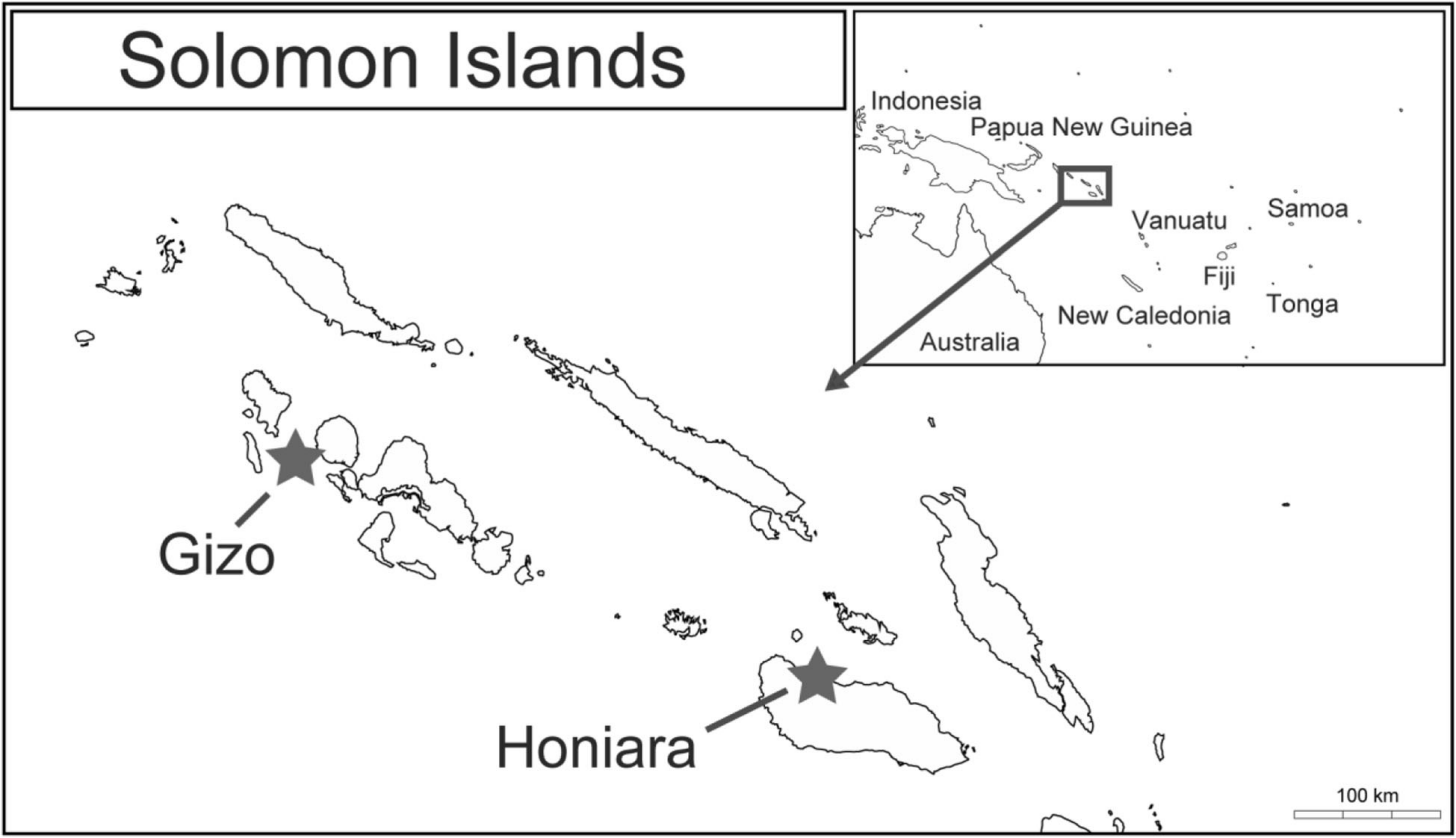
Map of Solomon Islands. A partial map of Solomon Islands, showing Honiara and Gizo, where the study was performed is shown

Solomon Islands is an archipelago, consisting of a double chain of islands, located in the southwest Pacific region. It has a population of more than half a million people. It shares a border with Papua New Guinea, and the densely populated region of SE Asia is located to the west (Fig. 1). While the majority of the population live in rural areas and are involved in subsistence agriculture, fast-growing unregulated urban settlements are also common.

The first reported DF outbreak in Solomon Islands occurred in Honiara, the capital city of Solomon Islands, in 1982, but it was only confirmed serologically in 1992 [11]. A 10-year inter-epidemic cyclic pattern was observed until 2008, when DENV-4 first started to circulate the region [12]. A subsequent outbreak in 2013 was more severe, resulting in the first reported deaths [13]. This outbreak was unique, as it lasted longer than usual, i.e., previous outbreaks lasted 2 years, but the 2013 outbreak lasted until early 2016. This, in addition to the global expansion and reported occurrence of Zika virus (ZIKV) and chikungunya virus (CHKV) infections in the region [14, 15], prompted us to carry out this study.

The study objectives were to estimate the seroprevalence of arboviruses in the urban population of Solomon Islands while using qPCR to determine the circulating viruses from the clinical samples; furthermore, to characterize and compare the dengue virus isolated from our study to previous published isolates from the region using the phylogenetic tree analysis.

## Materials and methods

### Data collection

Suspected cases of DF were reported syndromically as dengue-like illness (DLI), which was defined as the sudden onset of fever, testing negative for malaria, and exhibiting one or two clinical manifestations of DF, as described previously [16]. In order to obtain a complete picture, we only included the clinics in Honiara, including Gizo, that started reporting from 2013 to 2016. This ensured that we would be able to detect trends using consistent and complete datasets. The virological investigations involved 2-weeklong blood sample collection periods (in April or December 2016), during which blood samples were collected from DLI patients that visited the hospitals in Honiara and Gizo.

The household seroprevalence study was performed in April 2016 in Gizo (Western Province) and a community settlement (Tuvaruhu) that was representative of Honiara. Each of the subjects that gave their consent filled out a questionnaire, before approximately 500 μL of blood was collected in a pediatric tube, containing ethylenediaminetetraacetic acid (EDTA), using the finger prick method (Becton Dickinson collection system). The blood samples were brought back to the hospital laboratory and processed by centrifugation at 13000 rpm for 10 min to separate the plasma. The samples were then transported to our laboratory overseas, where they were stored at − 20 °C, before being used for various enzyme-linked immunosorbent assays tests.

### Serological tests

To determine the seroprevalence of arboviruses in the household study, tests for IgG antibodies against various arboviruses were performed using commercially available ELISA. These included tests for anti-DENV IgG antibodies (Vircell, Granada, Spain), anti-ZIKV IgG antibodies (MyBioSource Inc., CA, USA), and anti-CHIKV IgG antibodies (Abcam, Cambridge, UK). The controls provided with the kits and the cutoff values listed in the associated information were used to determine the results, as recommended by the manufacturers. The clinical samples from recent DLI cases were tested using non-structural protein-1 (NS1)/IgM/IgG combo rapid diagnostic test (RDT) kits for DENV. Tests for the other arboviruses were performed on selected December samples using commercial IgM ELISA kits (ZIKV IgM ELISA, MyBioSource Inc., and CHIKV IgM ELISA, Abcam).

### Molecular testing

The qPCR screening was performed using cDNA extracted from 200 μL of the serum samples from the patients with suspected DF. The cDNA was synthesized using the random primer protocol in the ReverTra Ace-α-® system (Toyobo, Osaka, Japan), as recommended by the manufacturer. The cDNA samples were stored at − 20 °C, before being used for various molecular tests. The qPCR screening used previously described DENV serotype-specific and ZIKV-specific primers (Table 1) with some modifications [17–19]. A 10-μL reaction mixture, containing 0.2 μM of each forward and reverse primer, 5 μL of 2× SsoAdvanced universal SYBR Green supermix taq (Bio-Rad Laboratories, Hercules, CA), and 1 μL template, was used together with the Rotor-Gene qPCR system (Qiagen, Hilden, Germany). The qPCR setup involved an initial temperature of 95 °C for 30 s, followed by 40 cycles of 15 s at 95 °C, and 30 s at 57 °C. To detect CHKV, the primers listed in Table 1 were designed based on the conserved region of the envelope gene, which was based on an alignment that included the Papua New Guinea (PNG) sequence (MF773569). The primer was first characterized by making 10-fold serial dilutions of 9.6 × 10^5^ copies of the E1 gene using the qPCR conditions described above. The amplification curve and *T*_m_ peak value were analyzed to calculate the correlation coefficient and consensus peak values of the primer. Sequence confirmation of 10% of the qPCR products was then performed to further validate the primer.

**Table 1:** The primers used in the qPCR screening of the arbo-viruses

	Forward primers	Reverse primers
DENV1	ATA CCY CCA ACA GCA GGA ATT	AGC ATR AGG AGC ATG GTC AC
DENV2	TGG ACC GAC AAA GAC AGA TTC TT	CGY CCY TGC AGC ATT CCA A
DENV3	AAG ACG GGA AACCG TCT ATC AA	TTG AGA ATC TCT TCG CCA ACT G
DENV4	CCA TCC CAC CRA CAG CAG G	CAA GAT GTT CAG CAT GCG GC
ZIKAV	AAG TTT GCA TGC TCC AAG AAA AT	CAG CAT TAT CCGGTA CTC CAG AT
CHIKV	CAA GAA AAT AAC ATC ACT GTA ACT	TCC AGG CTG AAG ACA TTG

### DENV envelope gene sequencing and phylogenetic tree analysis

The DENV envelope genes were investigated using cDNA templates from patients that were found to be positive for DENV in the clinical surveys conducted in Gizo and Honiara. The templates were amplified using TksGflex^TM^ DNA polymerase (TaKaRa Bio, Shiga, Japan) and previously described overlapping primers for DENV-2 and DENV-3 [20]. The PCR products were then cycle-sequenced using the BigDye Terminator cycle sequencing kit (version 3.1), before being sequenced on the ABI3130xl automated sequence analyzer (Thermo Fisher Scientific, Inc.). The final 1485-bp sequence for DENV-2 and the final 1479-bp sequence for DENV-3 were obtained by manually assembling the overlapping sequences in BioEdit (version 7.19) [21]. Our study sequences were uploaded to the DNA Data Bank of Japan (DDBJ) under accession numbers LC440471 to LC440484. These sequences were combined with those circulating SE Asia, Australia, and PICTs (from GenBank) to construct a phylogenetic tree using the maximum likelihood (ML) method in the software MEGA (version 7) [22]. Multiple full-envelope nucleotide sequences were aligned using Clustal W, which is integrated into the MEGA software, while evolutionary distances were computed using the recommended Tamura-Nei + G model. The reliability of the ML analysis was evaluated via a bootstrap test of 3000 replications. A different genotype from that of the interest group was set as an outgroup in each of the dengue trees.

### Data analysis

Descriptive statistical analyses were performed using the SPSS statistical software (version 18.0; SPSS Inc., IL, USA), and individual subjects were used as statistical units. Multinomial logistic regression analysis was performed to analyze the associations between selected variables and dengue infections. The prevalence rates for each variable were also determined, together with 95% confidence intervals (95% CI). A catalytic model with a constant force was used to calculate the force of infection (FOI), which was determined using the ML method, as described previously [23, 24].

## Results

### Honiara sentinel dengue reporting

**Fig. 2:**
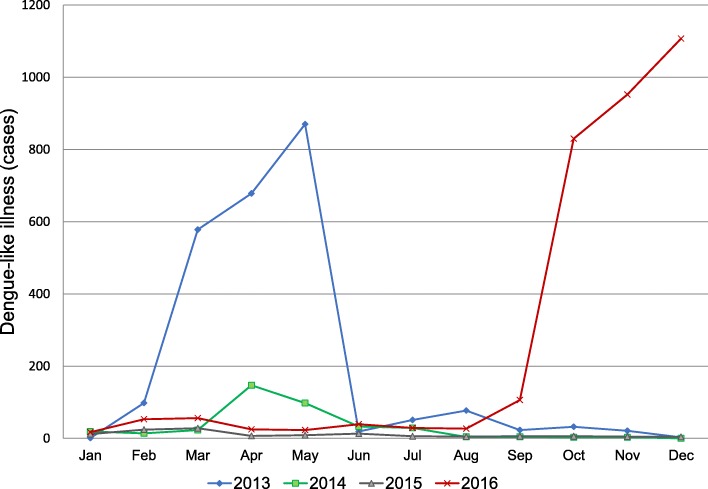
The number of cases detected in Honiara during the sentinel clinic surveillance from 2013 to 2016

The sentinel clinic data for DLI reported for Honiara, which covered the period from 2013 to 2016, are shown in Fig. 2. The graph shows that two outbreaks with very similar outbreak patterns occurred within 3 years, i.e., in 2013 and 2016. The outbreaks involved a sharp increase in the number of DLI cases in the first month and peaked in the subsequent 3 months. The 2016 sentinel data represented 45% of all DLI syndromic cases, as reported previously [16]. The 2016 outbreak differed from the previous outbreak in that it started in August rather than in January. Furthermore, the continuous persistent occurrence of DLI was seen before the spike in August 2016.

### Household survey results

The household survey involved 188 participants, who were recruited from Honiara (*n* = 78) or Gizo (*n* = 110). No refusals to participate were recorded in this study. There were slightly more females than males (male:female = 1:1.27). The subjects’ mean age was 21.1 years (95% CI 18.7–23.4; range: from 1 to 83 years old). The most common main source of income for the surveyed households was being employed in the private sector (43%), followed by being employed in the public sector (36%).

**Fig. 3:**
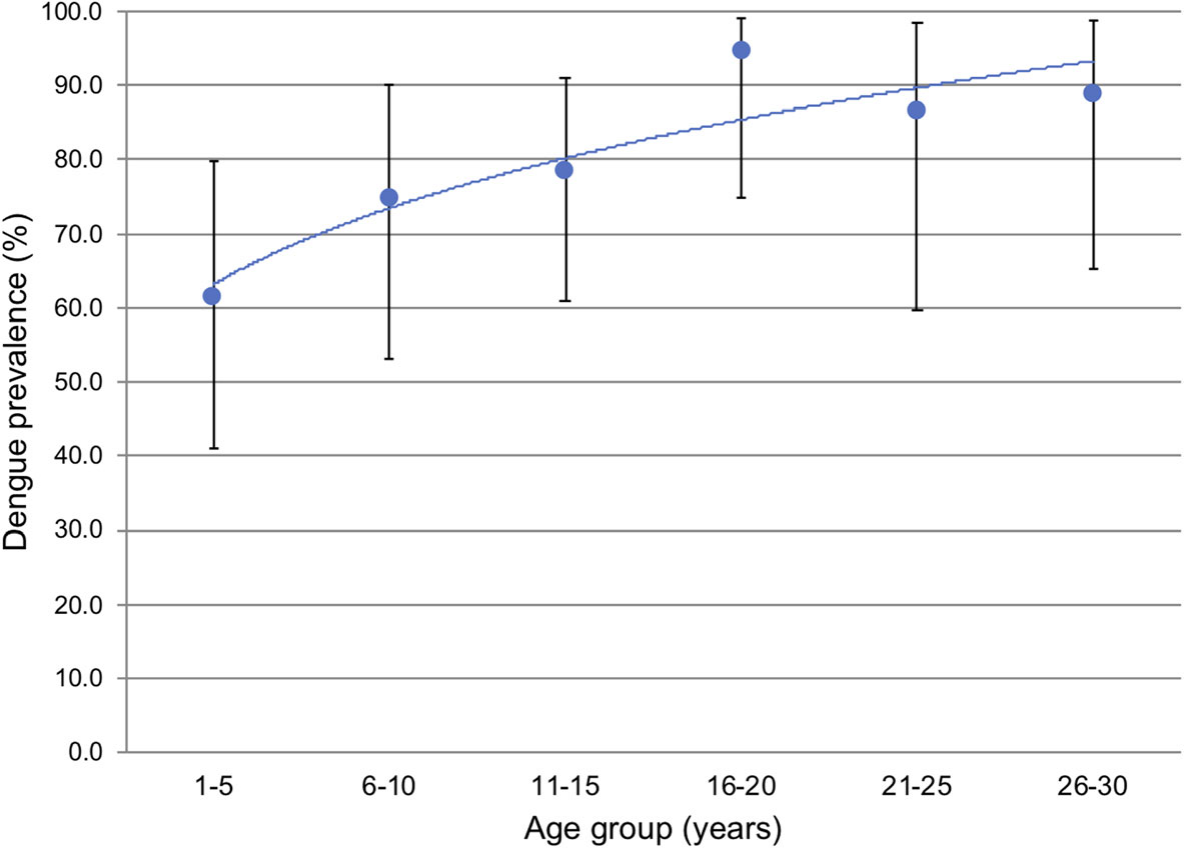
The prevalence of the DENV in different age groups in the household survey

The overall seroprevalence of the DENV, as measured by the rate of positive anti-DENV IgG antibody ELISA results, was 83.4% (95% CI 77.7–88.3%) (Table 2). The prevalence of the DENV was slightly higher in Honiara (88.4%) than in Gizo (80.0%), but the difference was not significant (*p* = 0.14). The prevalence of the DENV differed significantly between females (88.6%) and males (77.1%) (*p* value = 0.04). Neither the main household source of income nor recalled illnesses in the last 12 months significantly affected the prevalence of the DENV. The prevalence of the DENV increased with age, with the lowest prevalence rate (61.5%) seen in the 1–5 years age group and plateaued in the 16–20 years age group (Fig. 3). The mean ages of the subjects that tested positive and negative for anti-DENV IgG antibodies were 23.0 years and 11.7 years, respectively. The difference between the two groups (11.3 years) was significant. The FOI, which was defined as the annual risk of infection by any DENV serotype among DENV-naïve individuals, was 16.4% in the 1–20 years age group.

**Table 2:** The results of the household study in April 2016 showing the various arbovirus prevalence and characterization of the dengue infection in our study population

Variable	N	Positive (prevalence %)	95% CI (%)	*p* value
**A. Arboviruses**				
Dengue	188	158 (83.4)	(77.7–88.3)	
Zika	172	13 (7.6)	(4.1–11.6)	
Chikungunya	122	1 (0.9)	(0.0–8.6)	
**B. Dengue prevalence**				
**Site**				
Gizo	110	88 (80.0)	(71.3–87.0)	0.14
Honiara	78	69 (88.4)	(79.2–94.6)	
**Gender**				
Male	83	64 (77.1)	(66.6–85.6)	0.04
Female	105	93 (88.6)	(80.9–88.6)	
**Age group**				
1–5	26	16 (61.5)	(40.6–79.8)	0.00
6–10	24	18 (75)	(53.3–90.2)	0.02
11–15	33	26 (78)	(61.1–91.2)	0.04
16–20	20	19 (95)	(75.1–99.8)	0.90
**Main household source of income**				
Private sector	77	66 (85.7)	(75.9–92.0)	
Government	68	52 (76.5)	(64.6–85.9)	0.61
Self employed	23	21 (91.3)	(72.0–98.9)	0.16
Others	16	14 (87.5)	(61.5–98.5)	0.49
**12 months illness recall**				
Was ill	98	82 (83.7)	(74.8–90.3)	0.60
Had fever	69	54 (78.3)	(66.7–87.3)	0.07
Had malaria	56	48 (85.7)	(73.8–93.6)	0.36
Had dengue fever	15	13 (86.7)	(59.5–98.3)	0.64
**Force of infection**				
Age group 1–20	103	0.17	(0.135–0.231)	0.00

The prevalence results for the other arboviruses were 7.6% for the ZIKV and 0.9% for the CHKV (Table 2).

### Clinical survey results

The high positivity rate for anti-DENV IgG antibodies (77.9%) and the high DENV positivity rate detected in the qPCR analysis of the clinical samples (76.3%) were indicative of a high number of secondary infections in the 2016 outbreak. Our high prevalence results appeared to support the idea that IgG is a useful serological marker of past exposure to the DENV.

Among the 48 samples tested in April 2016, all of the detected DENV belonged to the DENV-3 serotype. In December, however, the dominant serotype detected in 131 tested samples was DENV-2 (74%), although 5 samples were co-infected with the DENV-3 serotype.

The qPCR-based ZIKV detection rate was 16.7% in April, and it increased to 41% in December, while the qPCR-based CHKV detection rate increased from 6.3% in April to 20.1% in December. Further analysis of the qPCR results showed that triple infections with DENV, ZIKV, and CHKV were detected in 15.8% of the December samples. Analysis of the December samples also revealed that there were only 4 CHKV-positive and 9 ZIKV-positive samples among the 34 DENV-negative samples.

### Sensitivity and specificity of the RDT for DENV

Among the clinical samples collected in December, a subset of 109 samples was used to calculate the sensitivity and specificity of the DENV RDT compared with the qPCR reference test (Table 3).

**Table 3:** Dengue RDT sensitivity and specificity on 109 clinical samples

Dengue assay	Sensitivity	Specificity	PPV	NPV
RDT NS1	49/82 (60%)	22/26 (84%)	49/53 (92%)	22/55 (40%)
RDT IgM or NS1	54/82 (66%)	18/26 (69%)	54/62 (87%)	18/46 (39%)
RDT IgG	26/82 (46%)	13/26 (50%)	26/39 (67%)	13/69 (19%)

The DENV RDT combo kit included tests for NS1, IgM, and IgG. The sensitivity and specificity of the NS1-based RDT were found to be 60% and 84%, respectively. When the NS1-based test was combined with the IgM-based test, the sensitivity of the RDT increased to 66%, but its specificity was reduced to 69%. The sensitivity and specificity of the IgG-based RDT were 46% and 50%, respectively, which the kit manufacturer’s information suggested was indicative of a high rate of secondary infections.

### DENV envelope gene phylogenetic analysis

#### Dengue virus serotype 3

**Fig. 4:**
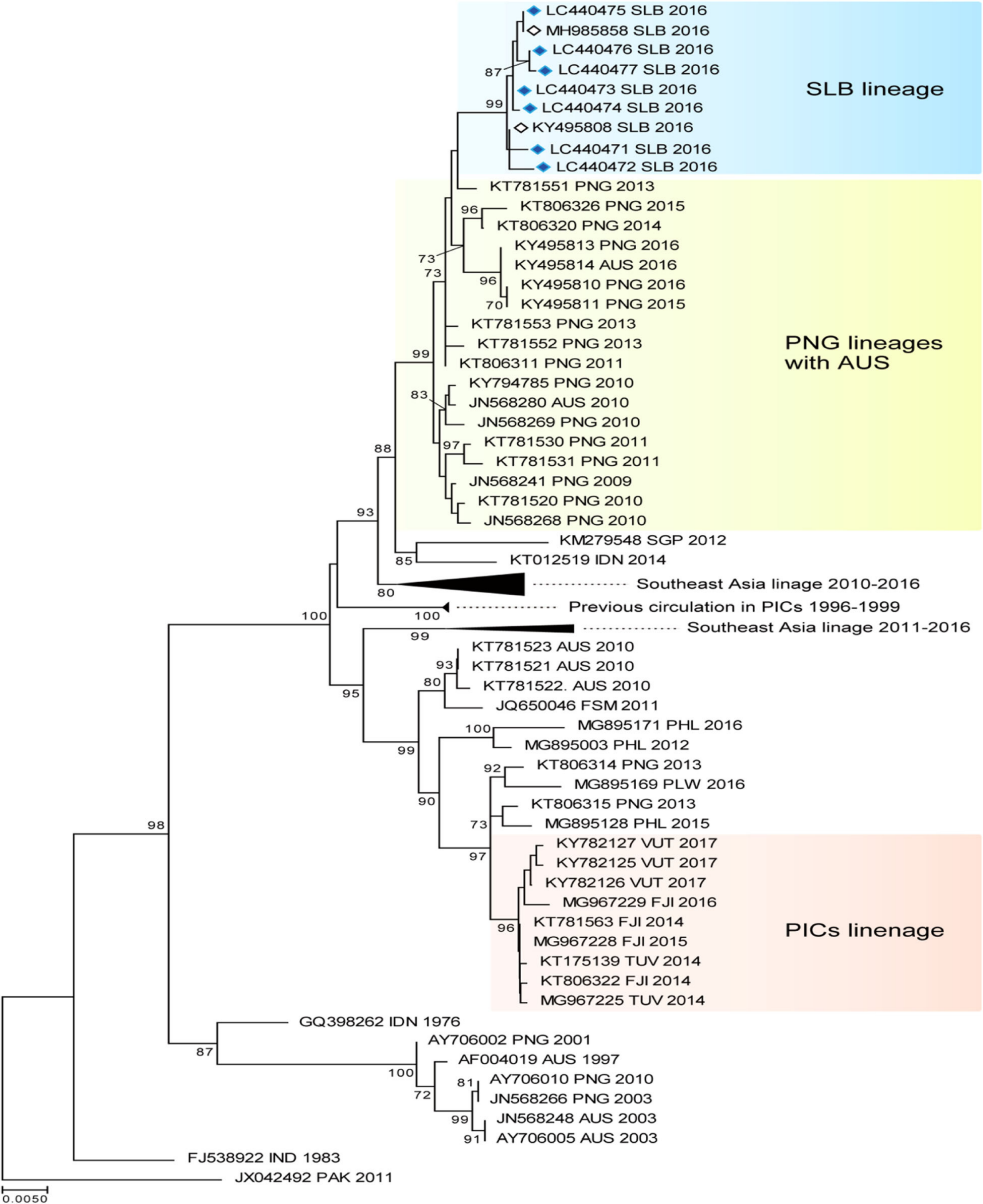
The evolutionary history of the DENV according to an analysis of DENV-3 envelope gene (1479-bp) sequences performed using the maximum likelihood method. The numbers for each node indicate bootstrap values (≥ 70%)

The phylogenetic tree for DENV-3 shown in Fig. 4 was constructed using 73 sequences from SE Asia, Australia, and the Pacific region.

The Solomon Island sequences were isolated from 2012 to 2016 and included 5 isolates from our study in April. The sequences in the tree were classed under DENV serotype 3, genotype-1. The PICT lineage, which included isolates from Solomon Islands, Fiji, Vanuatu, Samoa, and Nauru, shared a bootstrap value of 84 and circulated the region. They shared a common linkage with isolates from PNG (KT758738/2011 and KT758776/2014). An ancestral link with a bootstrap value of 95 to an isolate dating back to 2007 (KY794787/2007) was shared by all isolates found in PNG, Australia, and the PICTs. They also shared ancestral links with the isolates that had been circulating from 1989 to1996. The ancestral links also showed that SE Asia played an important role in the evolution of DF and its introduction to the PICTs.

#### Dengue virus serotype 2

**Fig. 5:**
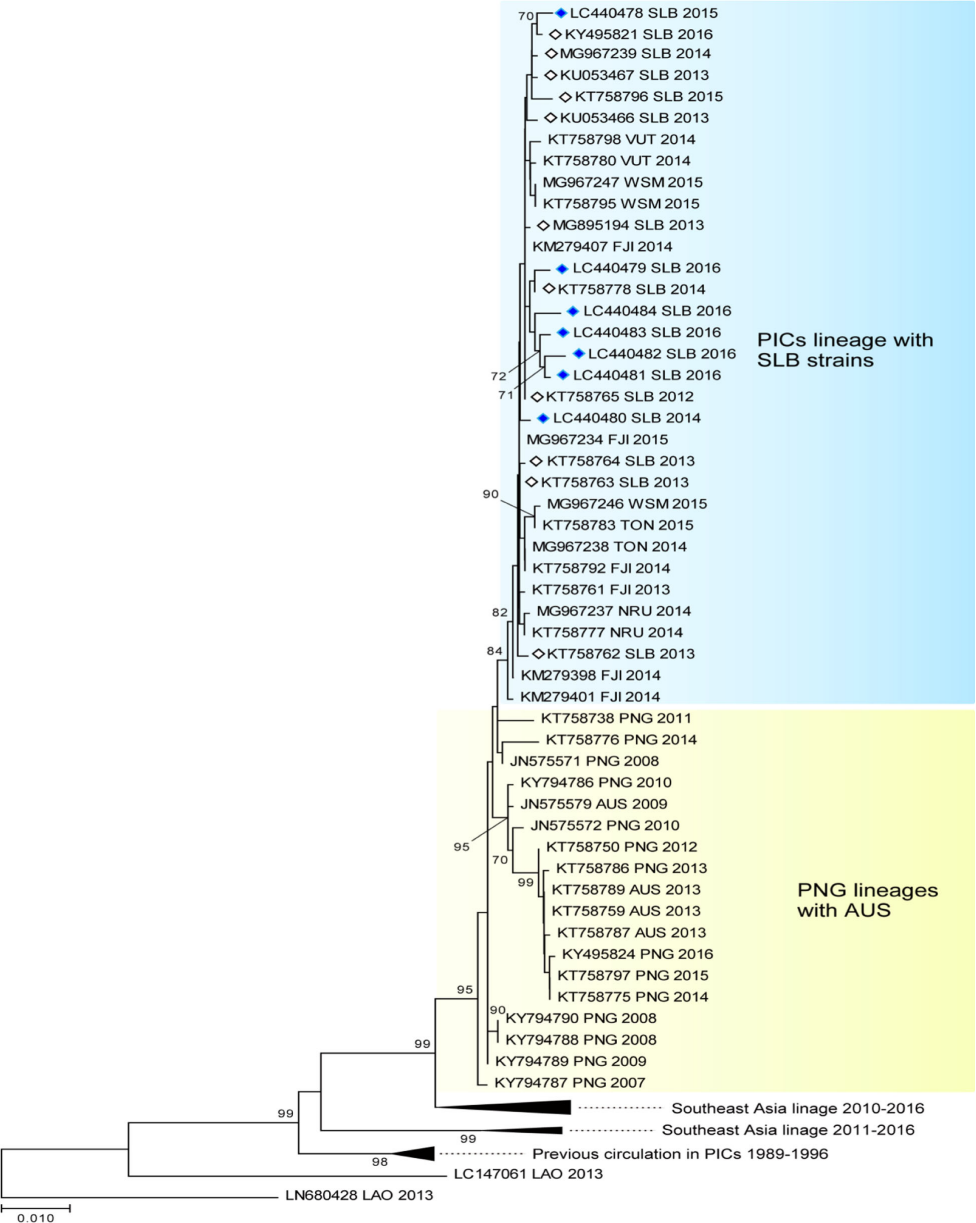
The evolutionary history of the DENV according to an analysis of DENV-2 envelope gene (1485-bp) sequences performed using the maximum likelihood method. The numbers for each node indicate bootstrap values (≥ 70%)

The DENV-2 phylogenetic tree shown in Fig. 5 was constructed using the 7 sequences we isolated in December and 75 sequences from SE Asia, Australia, and the PICTs, which were obtained from GenBank.

They were all classified as DENV-2, genotype-cosmopolitan. There were two distinct DENV-2 lineages circulating the Pacific region in 2016. The Solomon Island samples were all very similar and had a very strong bootstrap value of 99, which indicated that the virus was introduced at a single point and was circulating for a very short period of time. The lineage shared an ancestral link with a bootstrap value of 99 with the PNG/Australia lineages (2013–2016). The other PICT lineage, which had a bootstrap value of 96, circulated in Fiji, Tuvalu, and Vanuatu from 2014 to 2017 and shared an ancestral link with a bootstrap value of 99 with sequences from PNG/Australia (2010–2013). The geographical distribution of the virus, which included the northern Pacific region (FSM 2011 and Palau 2016), suggested that there were multiple entry routes, possibly including the Philippines (2015–2016). Only the Solomon Island lineage (2016) shared common ancestry with a virus that was previously circulating the Pacific region (1996–1999).

## Discussion

The prevalence of DENV in our study was comparable to those seen in other studies conducted in the region. For example, the prevalence of DENV was reported to be 95.6% in Samoa, 83% in PNG, and 83–96% in French Polynesia [9, 10, 25]. These values were similar to those reported in Asian countries: Bangladesh 80–83.3% [26], the Philippines 79.2% [27], and Thailand 79.2% [28].

Based on the syndromic data obtained in 2013, the high anti-DENV IgG antibody prevalence rate found in our study before the 2016 surge must have arisen during the first 3 months of an outbreak. This would have resulted in at least two-thirds of the population being exposed and becoming immune to the circulating serotype. A study conducted in Bangkok suggested that such exposure induces 3 years transient cross-protection against other DENV serotypes [29]. This would have provided herd immunity, which would have helped control the outbreak and could have been responsible for the alternative serotype outbreak pattern seen in the country [30]. It was also suggested that DENV-immune individuals are cross-protected against ZIKV [31], which would probably explain the low prevalence of ZIKV in April 2016 despite its circulation in the country in 2015 (GenBank KX 216632) [32].

The high qPCR-based DENV detection rate seen in our DENV-immune study population pointed to a high secondary DENV infection rate during the 2016 outbreak. Secondary infections involve heterologous DENV infections and are associated with an increased risk of severe DF [33, 34]. The DENV responsible for the new infection will trigger antibody production from pre-existing plasma cells, but these antibodies will not be able to neutralize the newly introduced serotype. The non-neutralizing binding of the antibodies to the virus results in the formation of a complex that binds to the Fcγ receptor, which is expressed on monocytes, macrophages, and dendritic cells, to facilitate antibody-dependent enhancement [35]. This process promotes virus production and an altered immune response, which is linked with severe DF.

The use of NS1-based DENV RDT in a resource-poor setting was very useful for diagnosis and management. However, the sensitivity value of the NS1-based RDT (60%) was low compared to the 95% sensitivity value reported in the previous DENV-3 outbreak in 2013 [36]. Secondary infections and the DENV serotype are important factors affecting the diagnostic sensitivity of NS1-based RDT [37, 38]. In our study, the NS1-based RDT was found to have lower sensitivity for the DENV-2 serotype than for the previously reported DENV-3.

The phylogenetic tree analysis and the serotype-specific qPCR results indicated that there was a single DENV-3 serotype in circulation from 2012 to 2016 in Solomon Islands. The newly introduced DENV-2 serotype in 2016 was responsible for the spike in cases seen in the latter part of 2016. This successful introduction was unique, as it was the first time that two serotypes were found to be co-circulating in the country. The co-circulation of multiple serotypes is a feature of endemic settings and was also recently reported in neighboring PNG [39]. The co-circulation of DENV-1 and DENV-4 has also been reported in New Caledonia [8], suggesting that such co-circulation is a potential threat in the region.

The presence of two lineages of the DENV-2 cosmopolitan serotype circulating the Pacific region in 2016 confirmed the heterogeneous nature of the virus [40] and the importance of monitoring the outbreak potential of different lineages. It also highlighted the importance of performing molecular sequencing analysis to identify genotype and subgenotype lineages. The DENV has probably been introduced into the PICTs multiple times from multiple sources. Our study, however, seemed to highlight ancestral linkages to PNG where the circulation of multiple serotypes and the formation of local lineages had been described [39]. Better molecular surveillance in PNG will enable more accurate prediction of DENV outbreaks in the Pacific region.

Recent improvements in airline travel links to the PICTs will make it easier to introduce new viruses into the region as airline travel was reported to increase the risk of DF spreading in Asia [41]. It is important for countries to have good surveillance mechanisms for both diseases and their vectors to enable timely interventions in future outbreaks.

### Strengths and limitations

The seroprevalence study was initially designed to explore the population’s immunity to DENV in order to predict its outbreak potential. The sample size was small, as it was based on the high DENV positivity rate (approximately 30%) seen during the last outbreak. It was therefore not ideal for investigating the ZIKV or CHKV, which have low prevalence rates. As the clinical study was initially only planned to involve molecular virological surveillance, it lacks demographic and clinical information. This also explains why the April 2016 samples were not subjected to serological testing. However, this study provided important information that will aid understanding of the unique outbreak pattern seen in this small population.

## Conclusion

This study found that the 2016 DLI outbreak in Solomon Islands was caused by multiple circulating arboviruses. The displacement of a single serotype (DENV-3) by a newly introduced one (DENV-2) was the predominant cause of the 2016 DLI outbreak, but there was evidence of ZIKV and CHKV co-circulating. The outbreak pattern suggests that natural population immunity is still the main control mechanism and that the population will always be vulnerable to vector-borne diseases. There is a need to step up vector-control activities and community mobilization to reduce the threat of such diseases in Solomon Islands. We recommend that Solomon Islands needs to increase its capacity for disease surveillance to include molecular screening of different arboviruses and some capacity to perform regular sequencing of the envelope genes of viral isolates. We also recommend the surveillance of disease vectors and their insecticide-resistance status. The communication of this information within the region will enable each country/territory to prepare as best as they can for the next outbreak.

## Data Availability

Not applicable

## Abbreviations

DF: Dengue fever
DLI: Dengue-like illness
ELISA: Enzyme-linked immunosorbent assays
DENV: Dengue virus
PICTs: Pacific Island countries
SE Asia: Southeast Asia
CHKV: Chikungunya virus
ZIKV: Zika virus
EDTA: Ethylenediaminetetraacetic acid
RDT: Rapid diagnostic test
PNG: Papua New Guinea
E1 gene: Envelope-1 gene
DDBJ: DNA Data Bank of Japan
ML: Maximum likelihood
FOI: Force of infection
HRC: Health Research Committee
