# Correction to: Multiple arboviral infections during a DENV-2 outbreak in Solomon Islands

**DOI:** 10.1186/s41182-020-00227-6

**Published:** 2020-06-11

**Authors:** Andrew Waleluma Darcy, Seiji Kanda, Tenneth Dalipanda, Cynthia Joshua, Takaki Shimono, Pheophet Lamaningao, Nobuyuki Mishima, Toshimasa Nishiyama

**Affiliations:** 1 Department of Hygiene and Public Health, Kansai Medical University, Hirakata, Japan; 2 Ministry of Health & Medical Services, Honiara, Solomon Islands

**Correction to: Trop Med Health 48, 33 (2020)**

**https://doi.org/10.1186/s41182-020-00217-8**

Following the publication of the original article [1], it was noted that Fig. 4 and Fig. 5 were exchanged. The figure legends for Fig. 4 and Fig. 5 are correct.

The correct figures have been included in this correction, and the original article has been corrected.

**Fig. 4:**
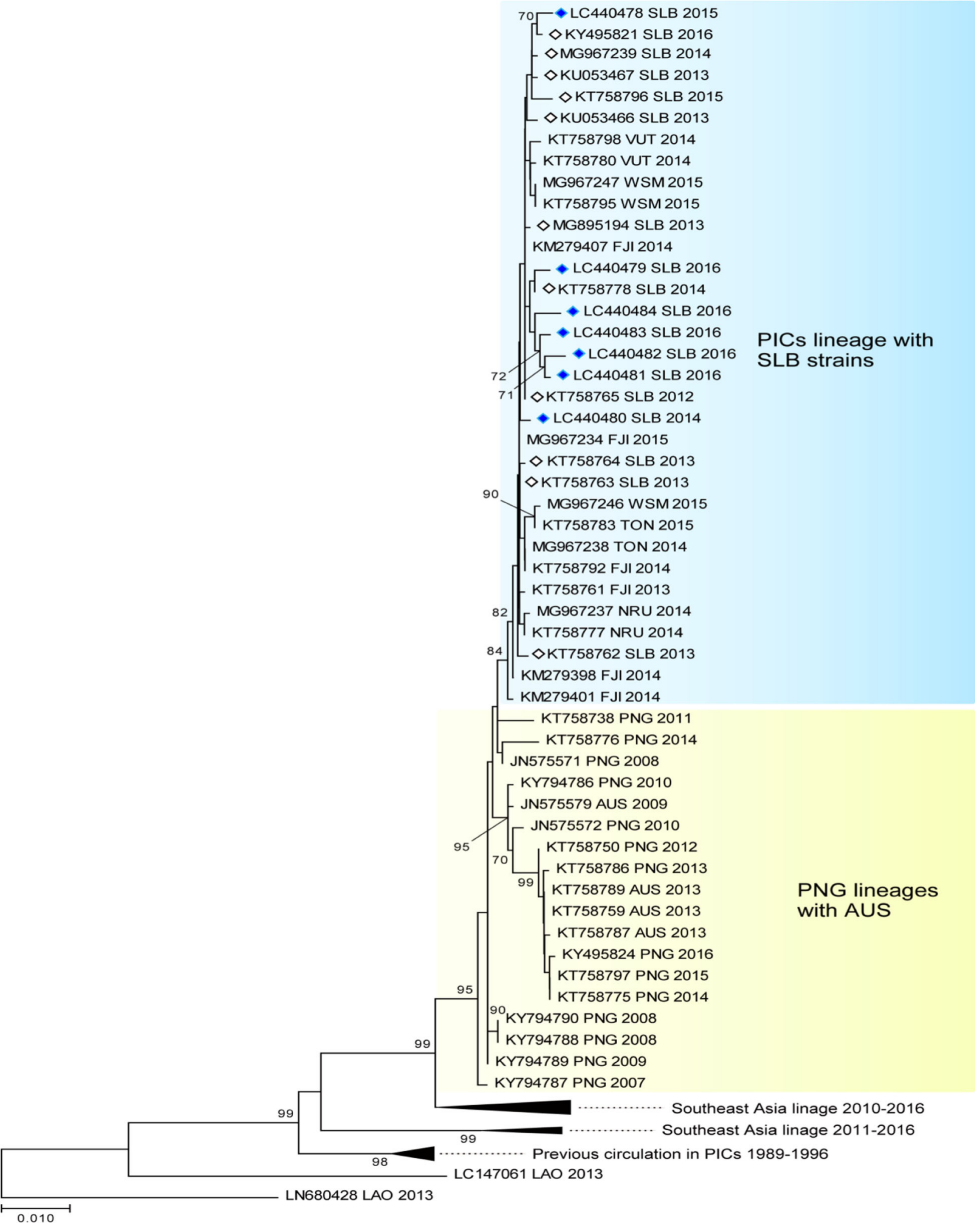
The evolutionary history of the DENV according to an analysis of DENV-3 envelope gene (1479-bp) sequences performed using the maximum likelihood method. The numbers for each node indicate bootstrap values (≥ 70%)

**Fig. 5:**
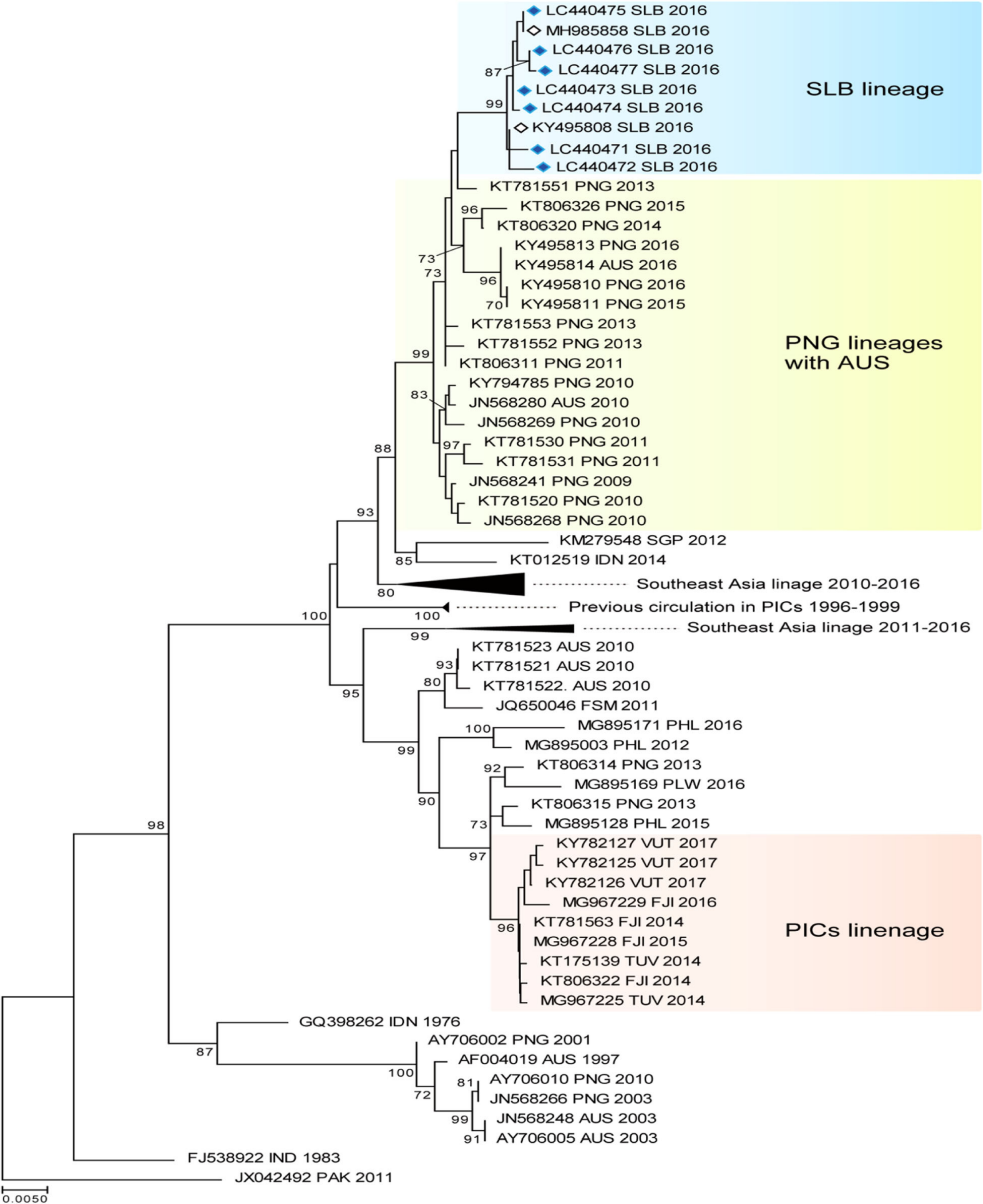
The evolutionary history of the DENV according to an analysis of DENV-2 envelope gene (1485-bp) sequences performed using the maximum likelihood method. The numbers for each node indicate bootstrap values (≥ 70%)
